# Bladder Neck Collagen Injection in the Treatment of Congenital Retrograde Ejaculation: A Case Report

**DOI:** 10.7759/cureus.1821

**Published:** 2017-11-03

**Authors:** Basri Cakiroglu, Orhun Sinanoglu, Ersan Arda

**Affiliations:** 1 Urology, Hisar Intercontinental Hospital; 2 Urology, Maltepe univers; 3 Urology, Trakya University

**Keywords:** retrograde ejaculation, bladder neck, collagen injection

## Abstract

The present study describes the first successful treatment of a congenital retrograde ejaculation case with a submucosal collagen injection to the bladder neck.

A 28-year-old male (height 170 cm, weight 80 kg) attended to the urology outpatient clinic with complaints of scattered urine stream and absence of ejaculation. The laboratory tests, including hormone profile (follicle* *stimulating hormone, luteinizing hormone, and testosterone) and the routine blood count were within normal ranges, whereas semen analysis demonstrated a total absence of ejaculation. The analysis of post-ejaculate urine specimens revealed an elevated sperm concentration indicative of retrograde ejaculation. The cystoscopy was performed; the prostatic urethra was normal, the verumontanum was in the orthotopic position, and the bladder neck was wide opened. Dextranomer/hyaluronic acid copolymer (Deflux, 8 ml) was injected into the bladder neck at clock positions of one, five, seven, and 11 o'clock. During the same procedure, the large opening in the vesical collum was obliterated. During the first follow-up (four weeks after surgery), the patient was able to produce a normal (2.8 ml) ejaculation volume. The sperm analysis revealed normozoospermia, with 25 million spermatozoa/ml, 26% (a) and 57% (a + b) motility, and 14% normal morphology.

The submucosal bladder neck collagen injection is a minimally invasive technique that quickly restores anterograde ejaculation and should be considered in the patients with congenital or acquired retrograde ejaculation and for those who did not respond to the medical treatment.

## Introduction

Retrograde ejaculation (RE) is a condition in which no or minimal antegrade ejaculation occurs, although orgasm is present and all the phases of ejaculation may have been felt by the patient [1]. It accounts not only for male infertility but also impaired sexual satisfaction. The etiology of RE may be congenital, acquired, or idiopathic in origin. The most frequent cause of acquired RE is prostatectomy, whereas congenital RE may be present in cases with posterior urethral valves and meatal stenosis or bladder neck incompetence due to exstrophy [2-3]. It may also occur in the patients with spina bifida. The medical treatment has been used to increase bladder neck tone but with limited efficacy, especially in a fixed anatomical deformity [4-5]. To our knowledge, we report the first successful treatment of RE due to a congenital cause with the transurethral submucosal injection of collagen to the bladder neck.

## Technical report

**Figure 1:**
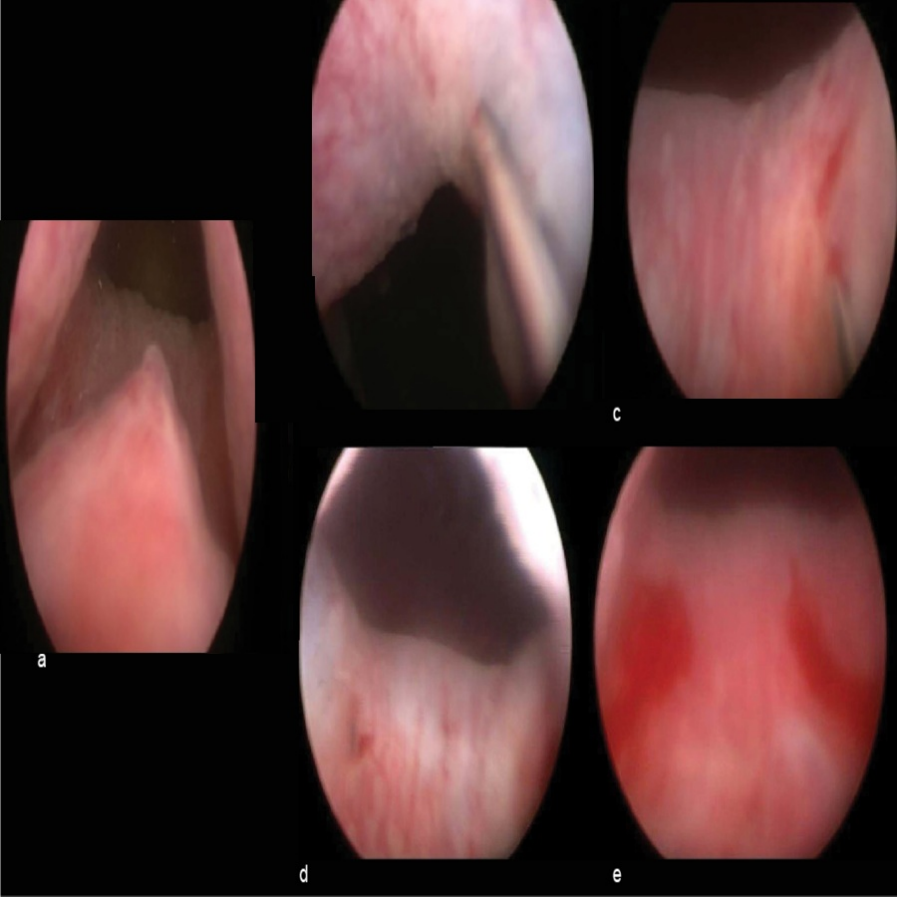
Figure representing the positions of the injections and the result. a: neutral position b: 1 o'clock position of the injection c: 5 o'clock position of the injection d: 7 o'clock position of the injection e: 11 o'clock position of the injection and the result.

A 28-year-old male (height 170 cm, weight 80 kg) attended to the urology outpatient clinic with the complaint of scattered urine stream and absence of ejaculation. His previous medical history has been normal, except for urine retention complaints, since his adolescence. On physical examination, both testes were in the scrotum with normal size and consistency, epididymal and ductal structures were normal, the penis was underdeveloped with ventrally hypospadic wide meatus, and the prostate was faintly palpable. The laboratory tests, including hormone profile (follicle stimulating hormone, luteinizing hormone, and testosterone) and routine blood count, were within normal ranges, whereas the semen analysis demonstrated a total absence of ejaculate. Post-ejaculate urine specimens contained a large number of sperms, indicating retrograde ejaculation. Initially, amitriptyline and oral pseudoephedrine were prescribed for four weeks with no positive effect on ejaculation. Subsequently, before the hypospadias repair, a cystoscopy was performed; the prostatic urethra was normal, verumontanum was in orthotopic position, and bladder neck was widely opened (Figure 1a). Nearly 8 ml of dextranomer/hyaluronic acid copolymer (Deflux) was injected into the bladder neck at positions one, five, seven, and 11 o'clock during the same session, which obliterated the large opening of the vesical collum (Figure 1 b-e).

During the first follow-up (four weeks after the surgery), the patient had a normal ejaculate (2.8 ml) after masturbation. The sperm analysis revealed 25 million spermatozoa/ml with 26% (a) and 57% (a + b) motility and 14% normal morphology (Table 1).

**Table 1:** Table representing the sperm analysis at fourth week. The sperm analysis at fourth week postoperative

	Ejaculate volume (ml)	Sperm count (milion/ml)	Morphology percent	Motility (a) percent	Motility (a+b) percent
Preoperative	0	0	0	0	0
Postoperative	2.8	25	14	26	57

## Discussion

The ejaculatory dysfunction is not frequent among infertility causes with an incidence rate of about 2% [6]. Its etiology is multifactorial, which can be acquired or congenital. The most frequent cause of acquired RE is prostatectomy. The bladder neck surgery during childhood is also one among the causes. Oral adrenergic agents have been used to increase the bladder neck tone but with limited efficacy, especially in a fixed anatomical deformity [4-5].

The congenital RE may be present in cases with posterior urethral valves and meatal stenosis or bladder neck incompetence due to exstrophy. It may also occur in the patients with spina bifida. Different congenital abnormalities can occasionally cause RE, e.g. ejaculatory ducts enter into the bladder and very rarely terminate the ectopic ureters or ectopic ureteroceles in the prostatic urethra [7]. For infertility issue, the bladder washing techniques with assisted reproductive techniques also have been successfully used, but they are labor intensive and do not treat the underlying bladder neck incompetence. To date, there are only two cases of collagen injection into the bladder neck in order to correct retrograde ejaculation, both of which were due to the acquired causes [8-9].

The first case underwent VY plasty in childhood, got partial benefit from antihistamines against RE, whereas, after collagen injection, there was normal semen ejaculation. The second RE case was due to the spinal trauma, causing urinary retention and RE has not benefited from imipramine hydrochloride and antihistamines, thus the antegrade ejaculation was ensured following total 6 cc collagen to the bladder neck at four, six, eight o’clock positions. In our case, 8 ml Deflux was injected at positions one, five, seven, and 11 o’clock.

## Conclusions

The aim of the collagen injection, in the present case, was to achieve antegrade ejaculation resulting in a satisfactory sexual relationship and also for the natural fertility. Minimal invasiveness, as well as the immediate efficacy of this technique, deserves consideration after the failure of the conservative treatments in patients with RE due to both congenital or acquired abnormalities.
